# A female case with novel KDM5C heterozygous variation presenting with Claes-Jensen type-like phonotype

**DOI:** 10.1186/s12883-022-03023-3

**Published:** 2022-12-19

**Authors:** Ruiyun Shen, Yanyang Li, Aiming Liang, Shijie Li, Chenlu Yang, Hongmei Huang

**Affiliations:** 1 Child Health Care Center, Beijing Children’s Hospital, Capital Medical University, National Center for Children’s Health, Beijing, 100045 China; 2 Pediatric Department, Huaihe Hospital of Henan University, Kaifeng, 475000 China

**Keywords:** KDM5C gene, X-linked disorder, Female, Case report, Phenotype

## Abstract

**Background:**

Lysine(K)-specific demethylase 5C (KDM5C) dysfunction causes X-linked syndromic intellectual developmental disorder Claes-Jensen type in male patients. The clinical presentations of female individuals with heterozygous KDM5C variations vary widely and are only now beginning to be characterized in detail.

**Case presentation:**

Herein, we identified a novel de novo heterozygous nonsense variation of KDM5C (c.3533C > A, p.S1178X) in a sporadic 4-year-old Chinese girl, who presented with Claes-Jensen type-like phenotypes, such as moderate developmental delay, serious expressive language delay, short stature, microcephaly, and typical facial particularities. Moreover, X-chromosome inactivation (XCI) analysis showed no significant skewed X-inactivation.

**Conclusion:**

The report expands the genotype of KDM5C variation in female patients, delineates the phenotype of affected females in this well-known X-linked disorder, and also reinforces the necessity to consider this X-linked gene, KDM5C, in sporadic female patients.

## Background

Located at Xp11.22, KDM5C contains 26 exons and encodes a ubiquitous 1560-aa protein, which is a versatile epigenetic regulator. It is crucial in the dose- and time-dependent control of cognitive development [1, 2]. KDM5C dysfunction causes X-linked intellectual disability in males (intellectual developmental disorder, X-linked, syndromic, Claes-Jensen type; MIM# 300,534), and is characterized by moderate-to-severe intellectual disability, short stature, dysmorphic features, seizures, and spasticity [3–5]. Most individuals with KDM5C variants are males; however, carrier females with milder phenotypes including intellectual disability and spasticity, have also been documented [1, 6, 7].

In a recent cohort study, approximately 70% of females heterozygous for pathogenic KDM5C variants were symptomatic, higher than 52% in another study [1, 7]. To date, all females with de novo mutation in the KDM5C gene have presented with symptomatic features [1]. The first de novo variant was reported in a female with spastic diplegia and speech dyspraxia [8]. Until now, appropriately eight affected females with de novo mutations have been documented [1, 5, 8, 9].

Here, we report a novel de novo variant (c.3533C > A, p.S1178X), a nonsense mutation of the KDM5C gene, identified in a 4-year-old girl who presented with global developmental delay, serious expressive language delay, short stature, and mild facial particularities.

## Case presentation

A 4-year-old girl with speech dyspraxia was referred to the Pediatric Developmental Behavior Clinic. She was full-term born to a woman aged > 40 years via vaginal delivery, G1P1, with a birth weight of 3100 g (75th percentile), birth height of 52.0 cm (75th percentile), and head circumference of 34.5 cm (50th percentile). No other medical issues or concerns were noted during the pregnancy period. She had been hospitalized twice during the neonatal period—once for pathological jaundice and once for sepsis. The proband could sit at the age of 6 months and walk at the age of 13 months; however, she had a significant expressive language delay. Her parents displayed normal physical growth and mental development, and both families declared no significant health or psychological issues.

On physical examination, her height was found to be 98 cm (3 percentiles for her age); weight, 15.2 kg (within the 3 to 10 percentiles for her age); and head circumference, 48.5 cm (< 3 percentiles for her age). Distinctive facial features, including a low-setting ear, bilateral nostril valgus, depressed nasal bridge, short philtrum, thin lips, and small chin, were noted. The Gesell Development Diagnosis Scale, which was used to assess the proband’s development quotient (DQ), reported the following findings: gross motor DQ 54, fine motor DQ 65, sociality DQ 54; and language DQ 39. The S-M social Living Ability Test resulted in a score of 8. During speech and body language communication in the consultation room, she displayed sharing behaviors or facial expressions and maintained normal back-and-forth conversations with normal eye contact.

His biochemical parameters, including serum or plasma levels of lactic acid; cysteine; very long chain fatty acids, amino acids; blood lipids; cholesterol; and hormones including thyroid stimulating hormone, thyroxine, luteinizing hormone, and follicle-stimulating hormone, were all within the normal range. No abnormal metabolites were noted in urinary mucopolysaccharides and organic acid, or on electroencephalography and head magnetic resonance imaging. Additionally, hearing and vision examination, echocardiography, abdominal ultrasound, gastrointestinal ultrasound, genitourinary ultrasound, and X-ray examination of bones revealed no abnormal findings.

**Fig. 1:**
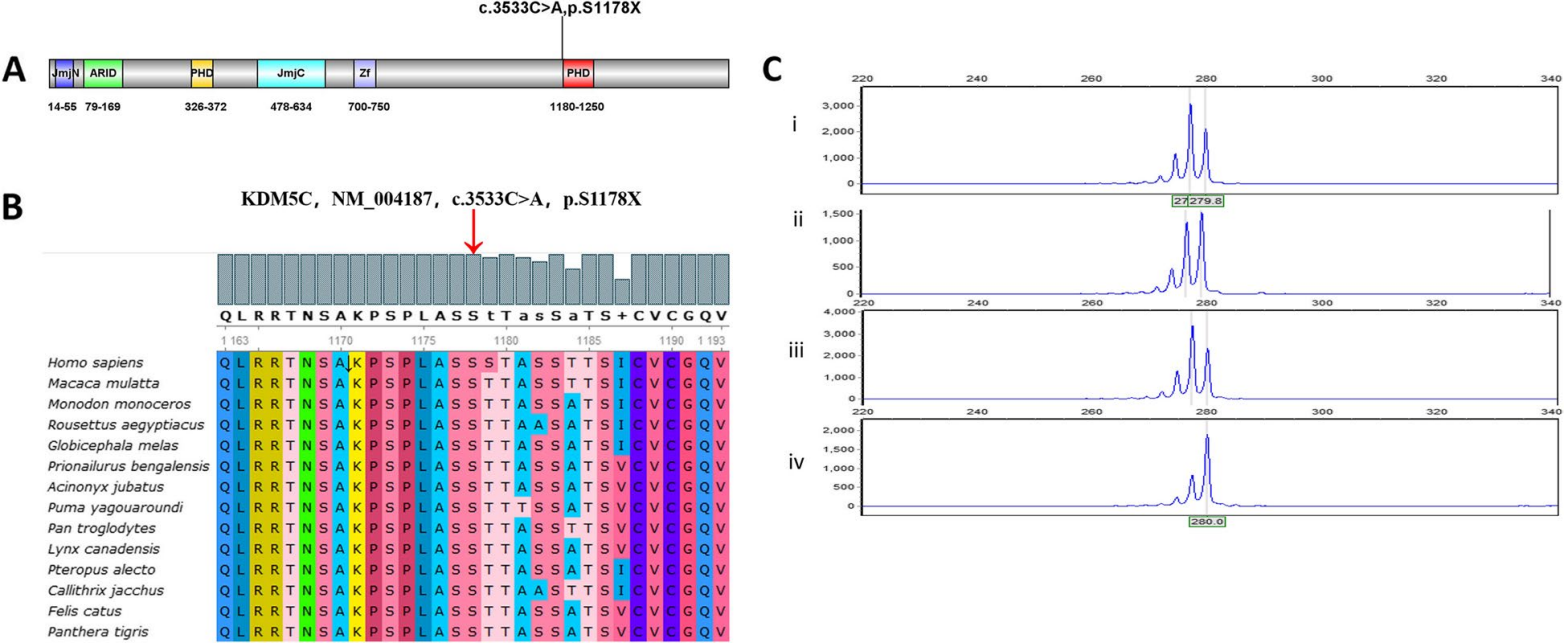
**A** Localization of this nonsense mutations (c.3533C > A, p.S1178X) on KDM5C. The variant was located 2 amino-acid upstream of the PHD2 domain. JmN, jumonji-N domain; ARID, AT-rich interacting domain; PHD, plant homeodomain box domain; JmjC, jumonji-C catalytic domain; ZF, zinc finger domain. **B** Multi-species conservation alignment of KDM5C homologs. The following RefSeq sequences were used for alignment: Homo sapiens NP_001340908.1, Macaca mulatta NP_001244296.1, Monodon monoceros XP_029096216.1, Rousettus aegyptiacus XP_036082315.1, Globicephala mela XP_030706894.1, Prionailurus bengalensis XP_043426298.1, Acinonyx jubatus XP_026909326.1, Puma yagouaroundi XP_040323006.1, Pan troglodytes XP_024209126.1, Lynx canadensis XP_030160481.1, Pteropus alecto XP_024896313.1, Callithrix jacchus XP_035144492.1, Felis catus XP_023105116.1, Panthera tigris XP_042829747.1. **C** X-chromosome inactivation analysis of the patient and her parents. i: PCR before digestion in the proband; ii: PCR after digestion in the proband; iii: PCR before digestion in her mother; iv: PCR before digestion in her father

Whole-exome sequencing was performed, which identified a de novo nonsense variant (c.3533C > A; p.S1178X) of the KDM5C gene in the proband (Fig. 1A); no other pathogenic gene variation was noted. This nonsense variation was expected to cause a loss-of-gene function; the proband’s parents lacked this variant. Serine 1178 is highly conserved across different species (Fig. 1B). This variation has not yet been reported in the literature or the ClinVar database, and is predicted to be “disease causing” by rare exome variant ensemble learner (REVEL). According to the American College of Medical Genetics and Genomics guidelines [10], this variation is apathogenic (PVS1 + PS2 + PM2).

X-chromosome inactivation (XCI) analysis was performed using the human androgen receptor assay as described previously [11]. Briefly, the genomic DNA was extracted from peripheral blood leukocytes and incubated with the restriction enzyme HpaII to digest the unmethylated active DNA. The mixture of digested and undigested DNA was PCR amplified with FAM-labelled fluorescent primers flanking a region comprising both the polymorphic and restriction sites, and then analyzed by capillary gel electrophoresis. When the X chromosome is inactivated, the restriction site of HpaII is methylated and cannot be cut, while the active X chromosome is the opposite. Therefore, if genomic DNA was digested first with HpaII followed by PCR amplification, only the inactivated X chromosome had a product. The degree of X-inactivation was calculated from the peak height. According to the analysis, the patient did not show significant skewed X-inactivation (Fig. 1C).

## Discussion and conclusions

We reported the case of a 4 years old Chinese girl with a de novo nonsense mutation (p.S1178X) in the KDM5C gene who exhibited the phenotypes of moderate gross developmental delay, serious language delay, short stature, microcephaly, and typical facial particularities. Her clinical phenotype was more severe than previously reported females, similar to Claes-Jensen type syndrome in males.

In our patient, the de novo nonsense variant (c.3533C > A; p.S1178X) in the KDM5C gene was a premature termination codon (PTC). When a stop codon is introduced, it causes nonsense-mediated decay of the mRNA harboring PTCs, resulting in very little protein production [12]. Thus, this new variant could be equivalent to a heterozygous null mutation. Approximately 30% of inherited human diseases are estimated to be due to the presence of PTCs or frameshifts that induce nonsense codons in mRNAs [13]. The site was highly conserved in different species, suggesting this nonsense mutation could cause functional damage. The C-terminal half of KDM5C contains a PHD finger domain (PHD2 domain) with an unknown function, which may recognize specific histone modifications and interact with other transcriptional regulators to recruit them to KDM5C-target genes [14]. Thus far, including the present case, only five variants in C-terminal segment have been described in detail, of which four (80%) were truncation mutations [1, 3, 15–18]. Although most of the functional domains were preserved in this truncated KDM5C, the level and stability were tremendously attenuated. The functional test identified that these truncation mutations resulted in loss-of-function of the KDM5C gene [19–21]. In a mouse model, Scandaglia et al. reported growth delay and memory deficits in female mice with heterozygous loss-of-function allele of KDM5C [22]. Patients with variants in the C-terminal segment have more severe intellectual disabilities than those with N-terminal segment variants, which may be associated with the presence of truncation mutations in most of them [21, 23]. It was reported before that the variants of distinct types locate preferentially around certain domains [23], but more evidence is needed to verify it. In the Human Gene Mutation Database (HGMD), more than 80 mutations in KDM5C gene have been identified, whereas not all of them may be pathogenic. Most of them fall within the N-terminal half of KDM5C— [21, 23–25]. The nonsense p.S1178X mutation in our proband was located in the 23rd exon, two amino acids upstream of the PHD2 domain (Fig. 1A).

In this study, we performed XCI analysis to identify any skewing that may have contributed to the phenotype, as noted in other diseases with an X-linked mode of inheritance. However, we found no significantly skewed XCI in our proband. Initially, phenotypic differences were attributed to XCI skewing in females with KDM5C variants. But with XCI analysis being conducted in more patients, no correlation was reported between the phenotype and XCI in females with KDM5C variants [1]. The KDM5C protein is broadly expressed in human brain tissue, neurons and astrocytes, and plays a key role in cognitive function [26, 27]. Their conserved co-expression in the human and mouse nervous systems suggested that the conserved regulatory mechanisms control the coordinated waves of gene expression during neurogenesis in specific brain areas [3, 19, 20, 28]. The inverted correlation between KDM5C and microRNA expression might contribute to the variable disease expressivity in brain tissue during development [1, 29]. Moreover, epigenetic modifications may modify the phenotype observed in females [30, 31].

In conclusion, this is the first report of a female with a de novo nonsense mutation at the C-terminal nonfunctional domain of KDM5C, which appeared to be causative of the Claes-Jensen type-like phenotype in our proband. Compared to previously reported females with de novo variants, our proband showed more severe features, notably expressive speech impairment, and dysmorphic features. These findings expand our understanding of the genotype of KDM5C variations in females with X-linked disease, and highlight the importance of considering the X-linked KDM5C gene in sporadic females during genetic counseling and medical management. Further studies are warranted to obtain the full picture of the genotype and phenotype of females with KDM5C variations.

## Data Availability

According to national legislation/guidelines, specifically the Administrative Regulations of the People’s Republic of China on Human Genetic Resources, the data of this project can be accessed with the accession code of CNP0003764 from the China National Genebank (CNGB, https://db.cngb.org/cnsa/) or the linkage: https://db.cngb.org/search/project/CNP0003764/.

## Abbreviations

KDM5C: Lysine(K)-specific demethylase 5C
XCI: X-chromosome inactivation
DQ: Development quotient
EEG: Electroencephalogram
MRI: Magnetic Resonance Imaging
PTCs: Premature termination codons
